# An extended reverse Hardy–Hilbert’s inequality in the whole plane

**DOI:** 10.1186/s13660-018-1706-y

**Published:** 2018-05-11

**Authors:** Qiang Chen, Bicheng Yang

**Affiliations:** 1 Department of Computer Science, Guangdong University of Education, Guangzhou, P.R. China; 2 Department of Mathematics, Guangdong University of Education, Guangzhou, P.R. China

**Keywords:** 26D15, Hardy–Hilbert’s inequality, Hermite–Hadamard’s inequality, Weight coefficient, Equivalent form, Reverse

## Abstract

Using weight coefficients, a complex integral formula, and Hermite–Hadamard’s inequality, we give an extended reverse Hardy–Hilbert’s inequality in the whole plane with multiparameters and a best possible constant factor. Equivalent forms and a few particular cases are considered.

## Introduction

If $p>1,\frac{1}{p}+\frac{1}{q}=1$, $a_{m},b_{n}\geq 0$, $0<\sum_{m=1}^{\infty }a_{m}^{p}<\infty $, $0<\sum_{n=1}^{\infty }b_{n}^{q}<\infty $, then we have the following Hardy–Hilbert inequality:

1 $$ \sum_{n=1}^{\infty }\sum _{m=1}^{\infty }\frac{a_{m}b_{n}}{m+n}< \frac{ \pi }{\sin (\frac{\pi }{p})}\Biggl( \sum_{m=1}^{\infty }a_{m}^{p} \Biggr)^{ \frac{1}{p}}\Biggl(\sum_{n=1}^{\infty }b_{n}^{q} \Biggr)^{\frac{1}{q}}, $$

with the best possible constant factor $\frac{\pi }{\sin (\pi /p)}$ [1]. A more accurate form of (1) with the same best possible constant factor was given in [2, Theorem 323]:

2 $$ \sum_{n=1}^{\infty }\sum _{m=1}^{\infty }\frac{a_{m}b_{n}}{m+n-1}< \frac{ \pi }{\sin (\frac{\pi }{p})}\Biggl( \sum_{m=1}^{\infty }a_{m}^{p} \Biggr)^{ \frac{1}{p}}\Biggl(\sum_{n=1}^{\infty }b_{n}^{q} \Biggr)^{\frac{1}{q}}. $$

Inequalities (1) and (2) played an important role in analysis and its applications (see [2–4]).

In 2011, Yang [5] gave the following an extension of (2): If $0<\lambda_{1}$, $\lambda_{2}\leq 1$, $\lambda_{1}+\lambda_{2}=\lambda $, $a_{m},b_{n}\geq 0$,

$$\begin{aligned}& \Vert a \Vert _{p,\varphi } = \Biggl\{ \sum_{m=1}^{\infty }(m- \alpha )^{p(1-\lambda_{1})-1}a _{m}^{p}\Biggr\} ^{\frac{1}{p}}\in (0, \infty ), \\& \Vert b \Vert _{q,\psi } = \Biggl\{ \sum_{n=1}^{\infty }(n- \alpha )^{q(1-\lambda_{2})-1}b _{n}^{q}\Biggr\} ^{\frac{1}{q}}\in (0, \infty ), \end{aligned}$$

then

3 $$ \sum_{n=1}^{\infty }\sum _{m=1}^{\infty }\frac{a_{m}b_{n}}{(m+n-2 \alpha )^{\lambda }}< B(\lambda_{1}, \lambda_{2})\Vert a \Vert _{p,\varphi }\Vert b \Vert _{q, \psi } \quad \biggl(0\leq \alpha \leq \frac{1}{2}\biggr), $$

where the constant factor $B(\lambda_{1},\lambda_{2})$ is the best possible, and $B(u,v)$ is the beta function defined as (see [6])

4 $$ B(u,v):= \int_{0}^{\infty }\frac{1}{(1+t)^{u+v}}t^{u-1}\,dt\quad (u,v>0). $$

For $\lambda =1$, $\lambda_{1}=\frac{1}{q}$, $\lambda_{2}=\frac{1}{p}$, and $\alpha =\frac{1}{2}$, (3) reduces to (2). Some other results related to (1)–(3) were provided in [7–24]. In 2016–17, a few extensions of (1)–(3) with some reverses in the whole plane were obtained in [25–27].

In this paper, using weight coefficients, a complex integral formula, and Hermite–Hadamard’s inequality, we give the following extension of the reverse of (1) in the whole plane: If $0< p<1$ ($q<0$), $\frac{1}{p}+\frac{1}{q}=1$, $0<\lambda_{1}$, $\lambda_{2}<1$, $\lambda_{1}+ \lambda_{2}=\lambda \leq 1$, $\xi ,\eta \in [ 0,\frac{1}{2}],a _{m},b_{n}\geq 0$,

$$\begin{aligned}& 0< \sum_{\vert m \vert =1}^{\infty }\vert m-\xi \vert ^{p(1-\lambda_{1})-1}a_{m}^{p}< \infty , \\& 0< \sum _{\vert n \vert =1}^{\infty }\vert n-\eta \vert ^{q(1-\lambda_{2})-1}b_{n}^{q}< \infty , \end{aligned}$$

then setting

5 $$\begin{aligned} \theta_{1}(\lambda_{2},m) &:=\frac{\lambda \sin (\frac{\pi \lambda _{1}}{\lambda })}{\pi } \int_{\frac{\vert m-\xi \vert }{1+\eta }}^{\infty }\frac{u ^{\lambda_{1}-1}}{u^{\lambda }+1}\,du \\ &=O \biggl( \frac{1}{\vert m-\xi \vert ^{\lambda_{2}}} \biggr) \in (0,1),\quad \vert m \vert \in \mathbf{N}, \end{aligned}$$

we have the following reverse Hilbert-type inequality in the whole plane:

6 $$\begin{aligned}& \sum_{\vert n \vert =1}^{\infty }\sum _{\vert m \vert =1}^{\infty }\frac{a_{m}b_{n}}{ \vert m-\xi \vert ^{\lambda }+ \vert n-\eta \vert ^{\lambda }} \\& \quad > \frac{2\pi }{\lambda \sin (\frac{\pi \lambda_{1}}{\lambda })} \Biggl[ \sum_{\vert m \vert =1}^{\infty } \bigl(1-\theta_{1}(\lambda_{2},m)\bigr)\vert m-\xi \vert ^{p(1-\lambda_{1})-1}a_{m}^{p} \Biggr] ^{\frac{1}{p}} \\& \quad \quad {}\times \Biggl[ \sum_{\vert n \vert =1}^{\infty }\vert n- \eta \vert ^{q(1-\lambda_{2})-1}b _{n}^{q} \Biggr] ^{\frac{1}{q}}. \end{aligned}$$

Moreover, we prove an extended inequality of (6) with multiparameters and a best possible constant factor. We also consider equivalent forms and a few particular cases.

## Some lemmas and an example

### Lemma 1

*Let* **C** *be the set of complex numbers*, $\mathbf{C}_{\infty }=\mathbf{C}\cup \{\infty \}$, *and let* $z_{k}\in \mathbf{C}\backslash \{z\mid \operatorname{Re}z\geq 0, \operatorname{Im}z=0\}$ ($k=1,2,\ldots ,n$) *be different points*. *Suppose that a function* $f(z)$ *is analytic in* $\mathbf{C}_{ \infty }$ *except for* $z_{i}$ ($i=1,2,\ldots ,n$) *and that* $z=\infty $ *is a zero point of* $f(z)$ *of order not less than *1. *Then*, *for* $\alpha \in \mathbf{R}$, *we have*

7 $$ \int_{0}^{\infty }f(x)x^{\alpha -1}\,dx= \frac{2\pi i}{1-e^{2\pi \alpha i}}\sum_{k=1}^{n} \operatorname{Re}s\bigl[f(z)z^{\alpha -1},z_{k}\bigr], $$

*where* $0<\operatorname{Im}\ln z=\arg z<2\pi $. *In particular*, *if* $z_{k}$ ($k=1,\ldots ,n$) *are all poles of order* 1, *then setting* $\varphi_{k}(z)=(z-z _{k})f(z)$ ($\varphi_{k}(z_{k})\neq 0$), *we have*

8 $$ \int_{0}^{\infty }f(x)x^{\alpha -1}\,dx= \frac{\pi }{\sin \pi \alpha } \sum_{k=1}^{n}(-z_{k})^{\alpha -1} \varphi_{k}(z_{k}). $$

### Proof

By [28] (p. 118) we have (7). We find

$$\begin{aligned} 1-e^{2\pi \alpha i} &=1-\cos 2\pi \alpha -i\sin 2\pi \alpha \\ &=-2i\sin \pi \alpha (\cos \pi \alpha +i\sin \pi \alpha ) \\ &=-2ie^{i \pi \alpha } \sin \pi \alpha . \end{aligned}$$

In particular, since $f(z)z^{\alpha -1}=\frac{1}{z-z_{k}}(\varphi_{k}(z)z ^{\alpha -1})$, it is obvious that

$$ \operatorname{Re}s\bigl[f(z)z^{\alpha -1},-z_{k} \bigr]=z_{k}{}^{\alpha -1}\varphi_{k}(z _{k})=-e^{i\pi \alpha }(-z_{k})^{\alpha -1} \varphi_{k}(z_{k}). $$

Then by (7) we obtain (8). □

### Example 1

For $s\in \mathbf{N=\{}1,2,\ldots \}$, $c_{s}\geq \cdots \geq c_{1}>0, \varepsilon >0$, $\lambda_{1},\lambda_{2}>0$, $\lambda_{1}+\lambda_{2}=s \lambda $, we define the function

$$ k_{s\lambda }(x,y)=\frac{1}{\prod_{k=1}^{s}(x^{\lambda }+c_{k}y^{ \lambda })} $$

and constants $\widetilde{c}_{k}=c_{k}+(k-1)\varepsilon $ ($k=1,\ldots ,s$).

Since $\widetilde{c}_{s}>\cdots >\widetilde{c}_{1}=c_{1}>0$, by (8) we find

$$\begin{aligned} \widetilde{k}_{s}(\lambda_{1}) &:=\int_{0}^{\infty }\frac{1}{\prod_{k=1}^{s}(t^{\lambda }+\widetilde{c}_{k})}t^{\lambda_{1}-1}\,dt \\ &\overset{u=t^{\lambda /s}}{=}\frac{1}{\lambda } \int_{0}^{\infty }\frac{1}{ \prod_{k=1}^{s}(u+\widetilde{c}_{k})}u^{\frac{\lambda_{1}}{\lambda }-1}\,du \\ &=\frac{\pi }{\lambda \sin (\frac{\pi \lambda_{1}}{\lambda })}\sum_{k=1}^{s} \widetilde{c}_{k}^{\frac{\lambda_{1}}{\lambda }-1} \prod_{j=1(j\neq k)}^{s} \frac{1}{\widetilde{c}_{j}-\widetilde{c}_{k}} \in \mathbf{R}_{+}. \end{aligned}$$

Since

$$\begin{aligned} 0 &< \widetilde{k}_{s}(\lambda_{1})=\frac{1}{\lambda } \int_{0}^{ \infty }\prod_{k=1}^{s} \frac{1}{u+\widetilde{c}_{k}}u^{\frac{\lambda _{1}}{\lambda }-1}\,du \\ &\leq \frac{1}{\lambda } \int_{0}^{\infty }\frac{1}{(u+c_{1})^{s}}u ^{\frac{\lambda_{1}}{\lambda }-1}\,du \\ &\overset{u=c_{1}v}{=}\frac{1}{\lambda c_{1}^{\lambda_{2}/\lambda }} \int_{0}^{\infty }\frac{1}{(v+1)^{s}}v^{\frac{\lambda_{1}}{\lambda }-1}\,dv \\ &=\frac{1}{\lambda c_{1}^{\lambda_{2}/\lambda }}B\biggl(\frac{\lambda_{1}}{ \lambda },\frac{\lambda_{2}}{\lambda }\biggr)\in \mathbf{R}_{+}, \end{aligned}$$

it follows that

9 $$\begin{aligned} k_{s}(\lambda_{1}) &=\lim_{\varepsilon \rightarrow 0^{+}} \widetilde{k}_{s}(\lambda_{1}) \\ &=\frac{\pi }{\lambda \sin (\frac{\pi \lambda_{1}}{\lambda })}\sum_{k=1}^{s}c_{k}^{\frac{\lambda_{1}}{\lambda }-1} \prod_{j=1(j\neq k)} ^{s}\frac{1}{c_{j}-c_{k}}\in \mathbf{R}_{+}. \end{aligned}$$

In particular, for $s=1$, we obtain

10 $$ k_{1}(\lambda_{1})=\frac{1}{\lambda } \int_{0}^{\infty }\frac{u^{( \lambda_{1}/\lambda )-1}}{u+c_{1}}\,du= \frac{\pi }{\lambda c_{1}^{ \lambda_{2}/\lambda }\sin (\frac{\pi \lambda_{1}}{\lambda })}; $$

for $c_{s}=\cdots =c_{1}$, we have

11 $$ k(\lambda_{1}):= \int_{0}^{\infty }\frac{t^{\lambda_{1}-1}}{(t^{\lambda }+c_{1})^{s}}\,dt= \frac{1}{\lambda c_{1}^{\lambda_{2}/\lambda }}B\biggl(\frac{ \lambda_{1}}{\lambda },\frac{\lambda_{2}}{\lambda }\biggr). $$

We further assume that $s\in \mathbf{N}$, $c_{s}\geq \cdots \geq c_{1}>0$, $\alpha ,\beta \in (0,\pi )$, $\xi ,\eta \in [ 0,\frac{1}{2}]$, $0<\lambda_{1},\lambda_{2},\lambda \leq 1,\lambda_{1}+\lambda_{2}=s \lambda $ ($s\geq 2$); $0<\lambda_{1}$, $\lambda_{2}<1$, $0<\lambda_{1}+\lambda_{2}=\lambda \leq 1$ ($s=1$). For $\vert t \vert >\frac{1}{2}$, we set

$$ A_{\zeta ,\theta }(t):=\vert t-\zeta \vert +(t-\zeta )\cos \theta $$

$((\zeta ,\theta ,t)=(\xi ,\alpha ,x)\mbox{ or }(\eta ,\beta ,y))$ and

$$\begin{aligned} k(x,y) &:=k_{s\lambda }\bigl(A_{\xi ,\alpha }(x),A_{\eta ,\beta }(y)\bigr) \\ &=\frac{1}{\prod_{k=1}^{s}(A_{\xi ,\alpha }^{\lambda }(x)+c_{k}A_{ \eta ,\beta }^{\lambda }(y))}. \end{aligned}$$

### Definition 1

Define the following weight coefficients:

12 $$\begin{aligned}& \omega (\lambda_{2},m) : =\sum_{\vert n \vert =1}^{\infty }k(m,n) \frac{A_{ \xi ,\alpha }^{\lambda_{1}}(m)}{A_{\eta ,\beta }^{1-\lambda_{2}}(n)},\quad \vert m \vert \in \mathbf{N}, \end{aligned}$$

13 $$\begin{aligned}& \varpi (\lambda_{1},n) : =\sum_{\vert m \vert =1}^{\infty }k(m,n) \frac{A_{ \eta ,\beta }^{\lambda_{2}}(n)}{A_{\xi ,\alpha }^{1-\lambda_{1}}(m)},\quad \vert n \vert \in \mathbf{N}, \end{aligned}$$

where $\sum_{\vert j \vert =1}^{\infty }\cdots =\sum_{j=-1}^{-\infty }+\cdots +\sum_{j=1}^{\infty }\cdots$ ($j=m,n$).

### Lemma 2

*With regards to the above agreement*, *replacing* $0<\lambda_{1}\leq 1$ ($0<\lambda_{1}<1$) *by* $\lambda_{1}>0$ *and setting*

$$ h_{\beta }(\lambda_{1}):=2k_{s}( \lambda_{1})\csc^{2}\beta , $$

*we still have*

14 $$ h_{\beta }(\lambda_{1}) \bigl(1-\theta (\lambda_{2},m) \bigr)< \omega (\lambda_{2},m)< h _{\beta }(\lambda_{1}),\quad \vert m \vert \in \mathbf{N,} $$

*where*

15 $$\begin{aligned} \theta (\lambda_{2},m) &:=\frac{1}{k_{s}(\lambda_{1})} \int_{\frac{A_{\xi ,\alpha }(m)}{(1+\eta )(1+\cos \beta )}}^{\infty }\frac{u ^{\lambda_{1}-1}}{\prod_{k=1}^{s}(u^{\lambda }+c_{k})}\,du \\ &=O \biggl( \frac{1}{A_{\xi ,\alpha }^{\lambda_{2}}(m)} \biggr) \in (0,1),\quad \vert m \vert \in \mathbf{N}. \end{aligned}$$

### Proof

For $\vert x \vert >\frac{1}{2}$, we set

$$\begin{aligned}& k^{(1)}(x,y) : =\frac{1}{\prod_{k=1}^{s}\{A_{\xi ,\alpha }^{\lambda }(x)+c_{k}[(y-\eta )(\cos \beta -1)]^{\lambda }\}}, \\& \quad y < -\frac{1}{2}, \\& k^{(2)}(x,y) : =\frac{1}{\prod_{k=1}^{s}\{A_{\xi ,\alpha }^{\lambda }(x)+c_{k}[(y-\eta )(1+\cos \beta )]^{\lambda }\}}, \\& \quad y > \frac{1}{2}, \end{aligned}$$

wherefrom, for $y>\frac{1}{2}$,

$$ k^{(1)}(x,-y)=\frac{1}{\prod_{k=1}^{s}\{A_{\xi ,\alpha }^{\lambda }(x)+c _{k}[(y+\eta )(1-\cos \beta )]^{\lambda }\}}. $$

We find

16 $$\begin{aligned}& \omega (\lambda_{2},m)=\sum_{n=-1}^{-\infty }k^{(1)}(m,n) \frac{A_{ \xi ,\alpha }^{\lambda_{1}}(m)}{[(n-\eta )(\cos \beta -1)]^{1-\lambda _{2}}} \\& \hphantom{\omega (\lambda_{2},m)=}{}+\sum_{n=1}^{\infty }k^{(2)}(m,n) \frac{A_{\xi ,\alpha }^{\lambda _{1}}(m)}{[(n-\eta )(1+\cos \beta )]^{1-\lambda_{2}}} \\& \hphantom{\omega (\lambda_{2},m)}{} = \frac{A_{\xi ,\alpha }^{\lambda_{1}}(m)}{(1-\cos \beta )^{1-\lambda _{2}}}\sum_{n=1}^{\infty } \frac{k^{(1)}(m,-n)}{(n+\eta )^{1-\lambda _{2}}} \\& \hphantom{\omega (\lambda_{2},m)=}{} +\frac{A_{\xi ,\alpha }^{\lambda_{1}}(m)}{(1+\cos \beta )^{1-\lambda _{2}}}\sum_{n=1}^{\infty } \frac{k^{(2)}(m,n)}{(n-\eta )^{1-\lambda _{2}}}. \end{aligned}$$

 □

It is evident that, for fixed $m\in \mathbf{N}$, $0<\lambda_{2}\leq 1$, $0< \lambda \leq 1$, both $\frac{k^{(1)}(m,-y)}{(y+\eta )^{1-\lambda_{2}}}$ and $\frac{k^{(2)}(m,y)}{(y- \eta )^{1-\lambda_{2}}}$ are strictly decreasing and strictly convex with respect to $y\in (\frac{1}{2},\infty )$ and satisfy

$$\begin{aligned}& \frac{k^{(i)}(m,(-1)^{i}y)}{[y-(-1)^{i}\eta ]^{1-\lambda_{2}}}>0, \\& \frac{d}{dy}\frac{k^{(i)}(m,(-1)^{i}y)}{[y-(-1)^{i}\eta ]^{1-\lambda _{2}}}< 0 \end{aligned}$$

and

$$ \frac{d^{2}}{dy^{2}}\frac{k^{(i)}(m,(-1)^{i}y)}{[y-(-1)^{i}\eta ]^{1- \lambda_{2}}}>0\quad (i=1,2). $$

By Hermite–Hadamard’s inequality (see [29]) we find

$$\begin{aligned} \omega (\lambda_{2},m) < &\frac{A_{\xi ,\alpha }^{\lambda_{1}}(m)}{(1- \cos \beta )^{1-\lambda_{2}}} \int_{\frac{1}{2}}^{\infty }\frac{k^{(1)}(m,-y)}{(y+ \eta )^{1-\lambda_{2}}}\,dy \\ &{}+\frac{A_{\xi ,\alpha }^{\lambda_{1}}(m)}{(1+\cos \beta )^{1- \lambda_{2}}} \int_{\frac{1}{2}}^{\infty }\frac{k^{(2)}(m,y)}{(y- \eta )^{1-\lambda_{2}}}\,dy. \end{aligned}$$

Setting $u=\frac{A_{\xi ,\alpha }(m)}{(y+\eta )(1-\cos \beta )}$ ($\frac{A_{\xi ,\alpha }(m)}{(y-\eta )(1+\cos \beta )}$) in the first (second) integral, by simplification we find

$$\begin{aligned} \omega (\lambda_{2},m) &< \biggl(\frac{1}{1-\cos \beta }+ \frac{1}{1+\cos \beta }\biggr) \int_{0}^{\infty }\frac{u^{\lambda_{1}-1}}{\prod_{k=1}^{s}(u ^{\lambda }+c_{k})}\,du \\ &=2k_{s}(\lambda_{1})\csc^{2}\beta =h_{\beta }(\lambda_{1}). \end{aligned}$$

Since both $\frac{k^{(1)}(m,-y)}{(y+\eta )^{1-\lambda_{2}}}$ and $\frac{k^{(2)}(m,y)}{(y-\eta )^{1-\lambda_{2}}}$ are strictly decreasing, we still have

$$\begin{aligned} \omega (\lambda_{2},m) &>\frac{A_{\xi ,\alpha }^{\lambda_{1}}(m)}{(1- \cos \beta )^{1-\lambda_{2}}} \int_{1}^{\infty }\frac{k^{(1)}(m,-y)}{(y+ \eta )^{1-\lambda_{2}}}\,dy \\ &\quad {}+\frac{A_{\xi ,\alpha }^{\lambda_{1}}(m)}{(1+\cos \beta )^{1- \lambda_{2}}} \int_{1}^{\infty }\frac{k^{(2)}(m,y)}{(y-\eta )^{1- \lambda_{2}}}\,dy \\ &=\frac{1}{1-\cos \beta } \int_{0}^{\frac{A_{\xi ,\alpha }(m)}{(1+ \eta )(1-\cos \beta )}}\frac{u^{\lambda_{1}-1}\,du}{\prod_{k=1}^{s}(u ^{\lambda }+c_{k})} \\ &\quad {}+\frac{1}{1+\cos \beta } \int_{0}^{\frac{A_{\xi ,\alpha }(m)}{(1- \eta )(1+\cos \beta )}}\frac{u^{\lambda_{1}-1}\,du}{\prod_{k=1}^{s}(u ^{\lambda }+c_{k})} \\ &\geq 2\csc^{2}\beta \int_{0}^{\frac{A_{\xi ,\alpha }(m)}{(1+\eta )(1+ \cos \beta )}}\frac{u^{\lambda_{1}-1}}{\prod_{k=1}^{s}(u^{\lambda }+c _{k})}\,du \\ &=h_{\beta }(\lambda_{2}) \bigl(1-\theta ( \lambda_{2},m)\bigr)>0, \end{aligned}$$

where $\theta (\lambda_{2},m)(<1)$ is indicated by (15). We obtain

$$\begin{aligned} 0 &< \theta (\lambda_{2},m)< \frac{1}{k_{s}(\lambda_{1})} \int_{\frac{A_{\xi ,\alpha }(m)}{(1+\eta )(1+\cos \beta )}}^{\infty }\frac{u ^{\lambda_{1}-1}}{u^{s\lambda }}\,du \\ &=\frac{1}{k_{s}(\lambda_{1})} \int_{\frac{A_{\xi ,\alpha }(m)}{(1+\eta )(1+\cos \beta )}}^{\infty }u ^{-\lambda_{2}-1}\,du \\ &=\frac{1}{\lambda_{2}k_{s}(\lambda_{1})} \biggl[ \frac{(1+\eta )(1+ \cos \beta )}{A_{\xi ,\alpha }(m)} \biggr] ^{\lambda_{2}}. \end{aligned}$$

Then we have (14) and estimate (15).  □

In the same way, we have

### Lemma 3

*With regards to the above agreement*, *replacing* $0<\lambda_{2}\leq 1$ ($0<\lambda_{2}<1$) *by* $\lambda_{2}>0$, *for*

$$ h_{\alpha }(\lambda_{1})=2k_{s}( \lambda_{1})\csc^{2}\alpha , $$

*we still have*

17 $$ h_{\alpha }(\lambda_{1}) \bigl(1-\vartheta ( \lambda_{1},n)\bigr)< \varpi (\lambda_{1},n)< h _{\alpha }(\lambda_{1}),\quad \vert n \vert \in \mathbf{N,} $$

*where*

18 $$\begin{aligned} \vartheta (\lambda_{1},n) &:=\frac{1}{k_{s}(\lambda_{1})} \int_{\frac{A_{\eta ,\beta }(n)}{(1+\xi )(1+\cos \alpha)}}^{\infty }\frac{u^{\lambda_{2}-1}}{\prod_{k=1}^{s}(u^{\lambda }+c_{k})}\,du \\ &=O \biggl( \frac{1}{A_{\eta ,\beta }^{\lambda_{1}}(n)} \biggr) \in (0,1),\quad \vert n \vert \in \mathbf{N}. \end{aligned}$$

### Lemma 4

*If* $\zeta \in {}[ 0,\frac{1}{2}],\theta \in (0,\pi )$, $(\zeta , \theta )=(\xi ,\alpha )$ (*or* $(\eta ,\beta )$), *then*, *for* $\rho >0$,

19 $$\begin{aligned} H_{\rho }(\zeta ,\theta )&:=\sum_{\vert k \vert =1}^{\infty } \frac{1}{A_{\zeta , \theta }^{1+\rho }(k)}=\frac{1+o(1)}{\rho } \\ &\quad {}\times \biggl[ \frac{1}{(1+\cos \theta )^{1+\rho }}+\frac{1}{(1-\cos \theta )^{1+\rho }} \biggr] \quad \bigl(\rho \rightarrow 0^{+}\bigr). \end{aligned}$$

### Proof

We find

$$\begin{aligned} H_{\rho }(\zeta ,\theta ) &=\sum_{k=-1}^{-\infty } \frac{1}{[(k- \zeta )(\cos \theta -1)]^{1+\rho }} \\ &\quad {}+\sum_{k=1}^{\infty }\frac{1}{[(k-\zeta )(\cos \theta +1)]^{1+ \rho }} \\ &=\frac{1}{(1-\cos \theta )^{1+\rho }}\sum_{k=1}^{\infty } \frac{1}{(k+ \zeta )^{1+\rho }} \\ &\quad {}+\frac{1}{(1+\cos \theta )^{1+\rho }}\sum_{k=1}^{\infty } \frac{1}{(k- \zeta )^{1+\rho }}. \end{aligned}$$

For $a=\frac{1}{(1-\zeta )^{1+\rho }}>0$, by Hermite–Hadamard’s inequality we have

$$\begin{aligned} H_{\rho }(\zeta ,\theta ) &\leq \biggl[ \frac{1}{(1-\cos \theta )^{1+ \rho }}+ \frac{1}{(1+\cos \theta )^{1+\rho }} \biggr] \\ &\quad {}\times \Biggl[ a+\sum_{k=2}^{\infty } \frac{1}{(k-\zeta )^{1+\rho }} \Biggr] \\ &< \biggl[ \frac{1}{(1-\cos \theta )^{1+\rho }}+\frac{1}{(1+\cos \theta )^{1+\rho }} \biggr] \\ &\quad {}\times \biggl[ a+ \int_{\frac{3}{2}}^{\infty }\frac{1}{(y-\zeta )^{1+ \rho }}\,dy \biggr] \\ &=\frac{a\rho +(\frac{3}{2}-\zeta )^{-\rho }}{\rho } \biggl[ \frac{1}{(1- \cos \theta )^{1+\rho }}+\frac{1}{(1+\cos \theta )^{1+\rho }} \biggr] . \end{aligned}$$

We still obtain

$$\begin{aligned}& H_{\rho }(\zeta ,\theta )\geq \biggl[ \frac{1}{(1-\cos \theta )^{1+ \rho }}+ \frac{1}{(1+\cos \theta )^{1+\rho }} \biggr] \sum_{k=1}^{\infty }\frac{1}{(k+\zeta )^{1+\rho }} \\& \hphantom{ H_{\rho }(\zeta ,\theta )}{}> \biggl[ \frac{1}{(1-\cos \theta )^{1+\rho }}+\frac{1}{(1+\cos \theta )^{1+\rho }} \biggr] \int_{1}^{\infty }\frac{dy}{(y+\zeta )^{1+ \rho }} \\& \hphantom{ H_{\rho }(\zeta ,\theta )}{}= \frac{(1+\zeta )^{-\rho }}{\rho } \biggl[ \frac{1}{(1-\cos \theta )^{1+ \rho }}+\frac{1}{(1+\cos \theta )^{1+\rho }} \biggr] . \end{aligned}$$

Hence we have (19). □

## Main results and some particular cases

### Theorem 5

*Suppose that* $0< p<1$ ($q<0$), $\frac{1}{p}+\frac{1}{q}=1$,

20 $$ K_{\alpha ,\beta }(\lambda_{1}):=h_{\beta }^{\frac{1}{p}}( \lambda_{1})h _{\alpha }^{\frac{1}{q}}(\lambda_{1})=2k_{s}( \lambda_{1}) \csc^{\frac{2}{p}}\beta \csc^{\frac{2}{q}}\alpha , $$

$a_{m},b_{n}\geq 0$ $(\vert m\vert ,\vert n\vert \in \mathbf{N})$, *and*

$$\begin{aligned}& 0< \sum_{\vert m \vert =1}^{\infty }A_{\xi ,\alpha }^{p(1-\lambda_{1})-1}(m)a_{m} ^{p}< \infty , \\& 0< \sum_{\vert n \vert =1}^{\infty }A_{\eta ,\beta }^{q(1-\lambda _{2})-1}(n)b_{n}^{q}< \infty . \end{aligned}$$

*We have the following reverse equivalent inequalities*:

21 $$\begin{aligned}& I:=\sum_{\vert n \vert =1}^{\infty }\sum _{\vert m \vert =1}^{\infty }\frac{1}{\prod_{k=1} ^{s}(A_{\xi ,\alpha }^{\lambda }(m)+c_{k}A_{\eta ,\beta }^{\lambda }(n))}a _{m}b_{n} \\& \hphantom{I}{}>K_{\alpha ,\beta }(\lambda_{1}) \Biggl[ \sum _{\vert m \vert =1}^{\infty }\bigl(1- \theta (\lambda_{2},m) \bigr)A_{\xi ,\alpha }^{p(1-\lambda_{1})-1}(m)a_{m} ^{p} \Biggr] ^{\frac{1}{p}} \Biggl[ \sum_{\vert n \vert =1}^{\infty }A_{\eta ,\beta }^{q(1-\lambda_{2})-1}(n)b _{n}^{q} \Biggr] ^{\frac{1}{q}}, \end{aligned}$$

22 $$\begin{aligned}& J : = \Biggl\{ \sum_{\vert n \vert =1}^{\infty }A_{\eta ,\beta }^{p\lambda_{2}-1}(n) \Biggl[ \sum_{\vert m \vert =1}^{\infty }\frac{a_{m}}{\prod_{k=1}^{s}(A_{\xi , \alpha }^{\lambda }(m)+c_{k}A_{\eta ,\beta }^{\lambda }(n))} \Biggr] ^{p} \Biggr\} ^{\frac{1}{p}} \\& \hphantom{J}{}> K_{\alpha ,\beta }(\lambda_{1}) \Biggl[ \sum _{\vert m \vert =1}^{\infty }\bigl(1- \theta (\lambda_{2},m) \bigr)A_{\xi ,\alpha }^{p(1-\lambda_{1})-1}(m)a_{m} ^{p} \Biggr] ^{\frac{1}{p}}, \end{aligned}$$

23 $$\begin{aligned}& L : = \Biggl\{ \sum_{\vert m\vert =1}^{\infty }\frac{A_{ \xi ,\alpha }^{q\lambda_{1}-1}(m)}{(1-\theta (\lambda_{2},m))^{q-1}} \Biggl[ \sum_{\vert n\vert =1}^{\infty } \frac{1}{ \prod_{k=1}^{s}(A_{\xi ,\alpha }^{\lambda }(m)+c_{k}A_{\eta ,\beta } ^{\lambda }(n))}b_{n} \Biggr] ^{q} \Biggr\} ^{\frac{1}{q}} \\& \hphantom{L}{}>K_{\alpha ,\beta }(\lambda_{1}) \Biggl[ \sum _{\vert n\vert =1} ^{\infty }A_{\eta ,\beta }^{q(1-\lambda_{2})-1}(n)b_{n}^{q} \Biggr] ^{\frac{1}{q}}. \end{aligned}$$

In particular, for $s=c_{1}=1$, $\alpha =\beta =\frac{\pi }{2}$ ($0<\lambda_{1}$, $\lambda_{2}<1$, $\lambda_{1}+\lambda_{2}=\lambda \leq 1$), (21) reduces to (6); and (22) and (23) reduce to the equivalent forms of (6) as follows:

24 $$\begin{aligned}& \Biggl\{ \sum_{\vert n \vert =1}^{\infty }\vert n-\eta \vert ^{p\lambda_{2}-1} \Biggl( \sum_{\vert m \vert =1}^{\infty } \frac{a_{m}}{\vert m-\xi \vert ^{\lambda }+\vert n-\eta \vert ^{ \lambda }} \Biggr) ^{p} \Biggr\} ^{\frac{1}{p}} \\& \quad > \frac{2\pi }{\lambda \sin (\frac{\pi \lambda_{1}}{\lambda })} \Biggl[ \sum_{\vert m \vert =1}^{\infty } \bigl(1-\theta_{1}(\lambda_{2},m)\bigr)\vert m-\xi \vert ^{p(1- \lambda_{1})-1}a_{m}^{p} \Biggr] ^{\frac{1}{p}}, \end{aligned}$$

25 $$\begin{aligned}& \Biggl[ \sum_{\vert m\vert =1}^{\infty } \frac{\vert m-\xi \vert ^{q \lambda_{1}-1}}{(1-\theta_{1}(\lambda_{2},m))^{q-1}} \Biggl( \sum_{\vert n\vert =1}^{\infty } \frac{b_{n}}{\vert m-\xi \vert ^{ \lambda }+\vert n-\eta \vert ^{\lambda }} \Biggr) ^{q} \Biggr] ^{\frac{1}{q}} \\& \quad > \frac{2\pi }{\lambda \sin (\frac{\pi \lambda_{1}}{\lambda })} \Biggl[ \sum_{\vert n\vert =1}^{\infty } \vert n-\eta \vert ^{q(1-\lambda_{2})-1}b _{n}^{q} \Biggr] ^{\frac{1}{q}}. \end{aligned}$$

### Proof

By the reverse Hölder inequality with weight (see [29]) and (12) we find

$$\begin{aligned}& \Biggl( \sum_{\vert m \vert =1}^{\infty }k(m,n)a_{m} \Biggr) ^{p} \\& \quad = \Biggl[ \sum_{\vert m \vert =1}^{\infty }k(m,n) \frac{A_{\xi ,\alpha }^{(1- \lambda_{1})/q}(m)a_{m}}{A_{\eta ,\beta }^{(1-\lambda_{2})/p}(n)}\frac{A _{\eta ,\beta }^{(1-\lambda_{2})/p}(n)}{A_{\xi ,\alpha }^{(1-\lambda _{1})/q}(m)} \Biggr] ^{p} \\& \quad \geq \sum_{\vert m \vert =1}^{\infty }h(m,n) \frac{A_{\xi ,\alpha }^{(1-\lambda_{1})p/q}(m)}{A _{\eta ,\beta }^{1-\lambda_{2}}(n)}a_{m}^{p} \Biggl[ \sum _{\vert m \vert =1}^{ \infty }k(m,n)\frac{A_{\eta ,\beta }^{(1-\lambda_{2})q/p}(n)}{A_{ \xi ,\alpha }^{1-\lambda_{1}}(m)} \Biggr] ^{p-1} \\& \quad = \frac{(\varpi (\lambda_{1},n))^{p-1}}{A_{\eta ,\beta }^{p\lambda_{2}-1}(n)} \sum_{\vert m \vert =1}^{\infty }k(m,n) \frac{A_{\xi ,\alpha }^{(1-\lambda_{1})p/q}(m)}{A _{\eta ,\beta }^{1-\lambda_{2}}(n)}a_{m}^{p}. \end{aligned}$$

By (17), in view of $p-1<0$, we have

26 $$\begin{aligned} J >&h_{\alpha }^{\frac{1}{q}}(\lambda_{1}) \Biggl[ \sum _{\vert n \vert =1}^{ \infty }\sum _{\vert m \vert =1}^{\infty }k(m,n)\frac{A_{\xi ,\alpha }^{(1-\lambda _{1})p/q}(m)}{A_{\eta ,\beta }^{1-\lambda_{2}}(n)}a_{m}^{p} \Biggr] ^{\frac{1}{p}} \\ =&h_{\alpha }^{\frac{1}{q}}(\lambda_{1}) \Biggl[ \sum _{\vert m \vert =1}^{\infty }\sum_{\vert n \vert =1}^{\infty }k(m,n) \frac{A_{\xi ,\alpha }^{(1-\lambda_{1})p/q}(m)}{A _{\eta ,\beta }^{1-\lambda_{2}}(n)}a_{m}^{p} \Biggr] ^{\frac{1}{p}} \\ =&h_{\alpha }^{\frac{1}{q}}(\lambda_{1}) \Biggl[ \sum _{\vert m \vert =1}^{\infty }\omega (\lambda_{2},m)A_{\xi ,\alpha }^{p(1-\lambda_{1})-1}(m)a_{m} ^{p} \Biggr] ^{\frac{1}{p}}. \end{aligned}$$

Then by (14) we have (22).

By Hölder’s inequality (see [29]) we have

27 $$\begin{aligned} I = \sum_{\vert n \vert =1}^{\infty } \Biggl[ A_{\eta ,\beta }^{\lambda_{2}- \frac{1}{p}}(n)\sum_{\vert m \vert =1}^{\infty }k(m,n)a_{m} \Biggr] A_{\eta , \beta }^{\frac{1}{p}-\lambda_{2}}(n)b_{n} \geq J \Biggl[ \sum_{\vert n \vert =1}^{\infty }A_{\eta ,\beta }^{q(1-\lambda_{2})-1}(n)b _{n}^{q} \Biggr] ^{\frac{1}{q}}. \end{aligned}$$

Then by (22) we have (21).

On the other hand, assuming that (21) is valid, we set

$$ b_{n}:=A_{\eta ,\beta }^{p\lambda_{2}-1}(n) \Biggl( \sum _{\vert m \vert =1}^{ \infty }k(m,n)a_{m} \Biggr) ^{p-1},\quad \vert n \vert \in \mathbf{N,} $$

and then

$$ J= \Biggl[ \sum_{\vert n \vert =1}^{\infty }A_{\eta ,\beta }^{q(1-\lambda_{2})-1}(n)b _{n}^{q} \Biggr] ^{\frac{1}{p}}. $$

By (26) we find $J>0$. If $J=\infty $, then (22) is evidently valid; if $J<\infty $, then by (21) we have

$$\begin{aligned}& \sum_{\vert n \vert =1}^{\infty }A_{\eta ,\beta }^{q(1-\lambda_{2})-1}(n)b_{n} ^{q} \\& \quad=J^{p}=I >K_{\alpha ,\beta }(\lambda_{1}) \Biggl[ \sum_{\vert m \vert =1}^{\infty }\bigl(1- \theta (\lambda_{2},m)\bigr)A_{ \xi ,\alpha }^{p(1-\lambda_{1})-1}(m)a_{m}^{p} \Biggr] ^{\frac{1}{p}} \Biggl[ \sum_{\vert n \vert =1}^{\infty }A_{\eta ,\beta }^{q(1-\lambda_{2})-1}(n)b _{n}^{q} \Biggr] ^{\frac{1}{q}}, \\& J = \Biggl[ \sum_{\vert n \vert =1}^{\infty }A_{\eta ,\beta }^{q(1-\lambda_{2})-1}(n)b _{n}^{q} \Biggr] ^{\frac{1}{p}} > K_{\alpha ,\beta }(\lambda_{1}) \Biggl[ \sum _{\vert m \vert =1}^{\infty }\bigl(1- \theta (\lambda_{2},m) \bigr)A_{\xi ,\alpha }^{p(1-\lambda_{1})-1}(m)a_{m} ^{p} \Biggr] ^{\frac{1}{p}}, \end{aligned}$$

namely, (22) follows, which is equivalent to (21).

We have proved that (21) is valid. Then we set

$$ a_{m}:= \frac{A_{\xi ,\alpha }^{q\lambda_{1}-1}(m)}{(1-\theta ( \lambda_{2},m))^{q-1}} \Biggl( \sum _{\vert n\vert =1}^{ \infty }k(m,n)b_{n} \Biggr) ^{q-1},\quad \vert m\vert \in \mathbf{N,} $$

and find

$$ L= \Biggl[ \sum_{\vert m\vert =1}^{\infty }\bigl(1-\theta ( \lambda_{2},m)\bigr)A _{\xi ,\alpha }^{p(1-\lambda_{1})-1}(m)a_{m}^{p} \Biggr] ^{\frac{1}{q}}. $$

If $L=0$, then (23) is impossible, so that $L>0$. If $L=\infty $, then (23) is trivially valid; if $L<\infty $, then we have

$$\begin{aligned}& \sum_{\vert m\vert =1}^{\infty }\bigl(1-\theta ( \lambda_{2},m)\bigr)A _{\xi ,\alpha }^{p(1-\lambda_{1})-1}(m)a_{m}^{p} \\& \quad =L^{q}=I>K_{\alpha ,\beta }(\lambda_{1}) \Biggl[ \sum _{\vert m\vert =1} ^{\infty }\bigl(1-\theta (\lambda_{2},m) \bigr)A_{\xi ,\alpha }^{p(1-\lambda_{1})-1}(m)a _{m}^{p} \Biggr] ^{\frac{1}{p}} \Biggl[ \sum_{\vert n\vert =1}^{\infty }A_{\eta , \beta }^{q(1-\lambda_{2})-1}(n)b_{n}^{q} \Biggr] ^{\frac{1}{q}}, \\& L = \Biggl[ \sum_{\vert m\vert =1}^{\infty }\bigl(1-\theta ( \lambda_{2},m)\bigr)A_{\xi ,\alpha }^{p(1-\lambda_{1})-1}(m)a_{m}^{p} \Biggr] ^{\frac{1}{q}} > K_{\alpha ,\beta }(\lambda_{1}) \Biggl[ \sum _{\vert n\vert =1}^{\infty }A_{\eta ,\beta }^{q(1- \lambda_{2})-1}(n)b_{n}^{q} \Biggr] ^{\frac{1}{q}}, \end{aligned}$$

thats is, (23) follows.

On the other-hand, assuming that (23) is valid, using the reverse Hölder inequality, we have

28 $$\begin{aligned} I =&\sum_{\vert m\vert =1}^{\infty } \biggl[ \frac{A_{ \xi ,\alpha }^{(1/q)-\lambda_{1}}(m)}{(1-\theta (\lambda_{2},m))^{-1/p}}a _{m} \biggr] \\ &\times \Biggl[ \frac{A_{\xi ,\alpha }^{\lambda_{1}-(1/q)}(m)}{(1- \theta (\lambda_{2},m))^{1/p}}\sum_{\vert n\vert =1}^{ \infty }k(m,n)b_{n} \Biggr] \\ \geq & \Biggl[ \sum_{\vert m\vert =1}^{\infty }\bigl(1- \theta ( \lambda_{2},m)\bigr)A_{\xi ,\alpha }^{p(1-\lambda_{1})-1}(m)a_{m}^{p} \Biggr] ^{\frac{1}{p}}L, \end{aligned}$$

and then by (23) we have (21), which is equivalent to (23).

Therefore, inequalities (21), (22), and (23) are equivalent. □

### Theorem 6

*With regards to the assumptions of Theorem *5, *the constant factor*$K _{\alpha ,\beta }(\lambda_{1})$ *in* (21), (22), *and* (23) *is the best possible*.

### Proof

For $0<\varepsilon <p\lambda_{1}$, we set $\widetilde{\lambda}_{1}=\lambda_{1}-\frac{\varepsilon }{p}$ ($\in (0,1)$), $\widetilde{\lambda }_{2}=\lambda_{2}+\frac{\varepsilon }{p}$ (>0), and

$$\begin{aligned}& \widetilde{a}_{m}:=A_{\xi ,\alpha }^{\lambda_{1}-(\varepsilon /p)-1}(m)=A _{\xi ,\alpha }^{\widetilde{\lambda }_{1}-1}(m)\quad \bigl(\vert m\vert \in \mathbf{N}\bigr), \\& \widetilde{b}_{n}:=A_{\eta ,\beta }^{\lambda_{2}-(\varepsilon /q)-1}(n)=A_{\eta ,\beta }^{\widetilde{\lambda }_{2}-\varepsilon -1}(n)\quad \bigl(\vert n\vert \in \mathbf{N}\bigr). \end{aligned}$$

By (19) and (17) we find

$$\begin{aligned}& \widetilde{I}_{1}:= \Biggl[ \sum_{\vert m\vert =1}^{\infty } \bigl(1- \theta (\lambda_{2},m)\bigr)A_{\xi ,\alpha }^{p(1-\lambda_{1})-1}(m) \widetilde{a}_{m}^{p} \Biggr] ^{\frac{1}{p}}\Biggl[ \sum_{\vert n\vert =1}^{\infty }A_{\eta , \beta }^{q(1-\lambda_{2})-1}(n) \widetilde{b}_{n}^{q} \Biggr] ^{ \frac{1}{q}} \\& \hphantom{\widetilde{I}_{1}} = \Biggl[ \sum_{\vert m\vert =1}^{\infty }A_{\xi ,\alpha } ^{-1-\varepsilon }(m)-\sum_{\vert m\vert =1}^{\infty }O\bigl(A _{\xi ,\alpha }^{-1-(\lambda_{2}+\varepsilon )}(m)\bigr) \Biggr] ^{ \frac{1}{p}} \Biggl( \sum _{\vert n\vert =1}^{\infty }A_{ \eta ,\beta }^{-1-\varepsilon }(n) \Biggr) ^{\frac{1}{q}} \\& \hphantom{\widetilde{I}_{1}} =\frac{1}{\varepsilon }\bigl(2\csc^{2}\alpha +o(1)-\varepsilon O(1) \bigr)^{ \frac{1}{p}}\bigl(2\csc^{2}\beta +\widetilde{o}(1) \bigr)^{\frac{1}{q}}\quad \bigl( \varepsilon \rightarrow 0^{+}\bigr), \\& \widetilde{I}:=\sum_{\vert m\vert =1}^{\infty } \sum _{\vert n\vert =1}^{\infty }k(m,n)\widetilde{a}_{m} \widetilde{b}_{n} \\& \hphantom{\widetilde{I}}=\sum_{\vert n\vert =1}^{\infty } \sum_{\vert m\vert =1}^{\infty }k(m,n)A_{\xi ,\alpha }^{ \widetilde{\lambda }_{1}-1}(m)A_{\eta ,\beta }^{\widetilde{\lambda }_{2}-\varepsilon -1}(n) \\& \hphantom{\widetilde{I}} = \sum_{\vert n\vert =1}^{\infty }\varpi ( \widetilde{ \lambda }_{1},n)A_{\eta ,\beta }^{-1-\varepsilon }(n)< h _{\alpha }( \widetilde{\lambda }_{1})\sum_{\vert n\vert =2} ^{\infty }A_{\eta ,\beta }^{-1-\varepsilon }(n) \\& \hphantom{\widetilde{I}} = \frac{1}{\varepsilon }h_{\alpha }\biggl(\lambda_{1}- \frac{\varepsilon }{p}\biggr) \bigl(2\csc^{2}\beta +\widetilde{o}(1) \bigr). \end{aligned}$$

If there exists a positive number $K\geq K_{\alpha ,\beta }(\lambda _{1})$ such that (21) is still valid when replacing $K_{\alpha ,\beta }(\lambda_{1})$ by *K*, then, in particular, we have

$$ \varepsilon \widetilde{I}=\varepsilon \sum_{\vert m\vert =1} ^{\infty }\sum_{\vert n\vert =1}^{\infty }k(m,n) \widetilde{a}_{m}\widetilde{b}_{n}>\varepsilon K \widetilde{I}_{1}. $$

In view of the preceding results, it follows that

$$\begin{aligned}& h_{\alpha }\biggl(\lambda_{1}-\frac{\varepsilon }{p}\biggr) \bigl(2\csc^{2}\beta + \widetilde{o}(1)\bigr) >K\cdot \bigl(2\csc^{2}\alpha +o(1)-\varepsilon O(1) \bigr)^{1/p}\bigl(2\csc^{2} \beta +\widetilde{o}(1) \bigr)^{1/q}, \end{aligned}$$

and then

$$ 4k_{s}(\lambda_{1})\csc^{2}\alpha\csc^{2}\beta \geq 2K\csc^{2/p} \alpha \csc^{2/q}\beta \quad \bigl(\varepsilon \rightarrow 0^{+}\bigr), $$

namely,

$$ K_{\alpha ,\beta }(\lambda_{1})=2k_{s}(\lambda_{1})\csc^{2/p}\beta \csc^{2/q}\alpha \geq K. $$

Hence $K=K_{\alpha ,\beta }(\lambda_{1})$ is the best possible constant factor in (21).

The constant factor $K_{\alpha ,\beta }(\lambda_{1})$ in (22) ((23)) is still the best possible. Otherwise, we would reach a contradiction by (27) ((28)) that the constant factor in (21) is not the best possible. □

### Remark 1

(i) For $\xi =\eta =0$ and $\alpha =\beta =\frac{\pi }{2}$ in (21), setting

$$\begin{aligned} \widetilde{\theta }(\lambda_{2},m) &:=\frac{1}{k_{s}(\lambda_{1})} \int_{\vert m \vert }^{\infty }\frac{u^{\lambda_{1}-1}}{\prod_{k=1}^{s}(u^{ \lambda }+c_{k})}\,du \\ &=O \biggl( \frac{1}{\vert m \vert ^{\lambda_{2}}} \biggr) \in (0,1),\quad \vert m \vert \in \mathbf{N}, \end{aligned}$$

we have the following new reverse inequality with the best possible constant factor $2k_{s}(\lambda_{1})$:

29 $$\begin{aligned}& \sum_{\vert n \vert =1}^{\infty }\sum _{\vert m \vert =1}^{\infty }\frac{1}{\prod_{k=1} ^{s}(\vert m \vert ^{\lambda }+c_{k}\vert n \vert ^{\lambda })}a_{m}b_{n} \\& \quad >2k_{s}( \lambda _{1}) \Biggl[ \sum_{\vert m \vert =1}^{\infty }\bigl(1- \widetilde{\theta }(\lambda_{2},m)\bigr)\vert m \vert ^{p(1- \lambda_{1})-1}a_{m}^{p} \Biggr] ^{\frac{1}{p}} \Biggl[ \sum _{\vert n \vert =1}^{ \infty }\vert n \vert ^{q(1-\lambda_{2})-1}b_{n}^{q} \Biggr] ^{\frac{1}{q}}. \end{aligned}$$

It follows that (21) is an extension of (29).

(ii) If $a_{-m}=a_{m}$ and $b_{-n}=b_{n}$ ($m,n\in \mathbf{N}$), then for

$$\begin{aligned} \widetilde{\theta }(\lambda_{2},m) =&\frac{1}{k_{s}(\lambda_{1})} \int _{m}^{\infty }\frac{u^{\lambda_{1}-1}}{\prod_{k=1}^{s}(u^{\lambda }+c _{k})}\,du \\ =&O \biggl( \frac{1}{m^{\lambda_{2}}} \biggr) \in (0,1),\quad m\in \mathbf{N}, \end{aligned}$$

(29) reduces to the following reverse Hilbert-type inequality:

30 $$\begin{aligned}& \sum_{n=1}^{\infty }\sum _{m=1}^{\infty }\frac{1}{\prod_{k=1}^{s}(m ^{\lambda }+c_{k}n^{\lambda })}a_{m}b_{n} \\& \quad > k_{s}(\lambda_{1}) \Biggl[ \sum _{m=1}^{\infty }\bigl(1- \widetilde{\theta }( \lambda_{2},m)\bigr)m^{p(1-\lambda_{1})-1}a_{m}^{p} \Biggr] ^{\frac{1}{p}} \Biggl[ \sum_{n=1}^{\infty }n^{q(1-\lambda_{2})-1}b_{n} ^{q} \Biggr] ^{\frac{1}{q}}. \end{aligned}$$

(iii) If $a_{-m}=a_{m}$ and $b_{-n}=b_{n}$ ($m,n \in \mathbf{N}$), then setting

$$\begin{aligned} \theta_{2}(\lambda_{2},m) :=&\frac{\lambda \sin (\frac{\pi \lambda _{1}}{\lambda })}{\pi } \int_{\frac{m-\xi }{1+\eta }}^{\infty }\frac{u ^{\lambda_{1}-1}}{u^{\lambda }+1}\,du \\ =&O \biggl( \frac{1}{(m-\xi )^{\lambda_{2}}} \biggr) \in (0,1),\quad m\in \mathbf{N}, \\ \theta_{3}(\lambda_{2},m) :=&\frac{\lambda \sin (\frac{\pi \lambda _{1}}{\lambda })}{\pi } \int_{\frac{m+\xi }{1+\eta }}^{\infty }\frac{u ^{\lambda_{1}-1}}{u^{\lambda }+1}\,du \\ =&O \biggl( \frac{1}{(m+\xi )^{\lambda_{2}}} \biggr) \in (0,1),\quad m\in \mathbf{N}, \end{aligned}$$

(6) reduces to

31 $$\begin{aligned}& \sum_{n=1}^{\infty }\sum _{m=1}^{\infty } \biggl[ \frac{1}{(m-\xi )^{ \lambda }+(n-\eta )^{\lambda }}+ \frac{1}{(m-\xi )^{\lambda }+(n+ \eta )^{\lambda }} \\& \quad \quad {}+\frac{1}{(m+\xi )^{\lambda }+(n-\eta )^{\lambda }}+\frac{1}{(m+ \xi )^{\lambda }+(n+\eta )^{\lambda }} \biggr] a_{m}b_{n} \\& \quad > \frac{2\pi }{\lambda \sin (\pi \lambda_{1}/\lambda )} \Biggl\{ \sum_{m=1}^{\infty } \bigl[ \bigl(1-\theta_{2}(\lambda_{2},m)\bigr) (m-\xi )^{p(1- \lambda_{1})-1} \\& \quad \quad {} +\bigl(1-\theta_{3}(\lambda_{2},m) \bigr) (m+\xi )^{p(1-\lambda_{1})-1} \bigr] a _{m}^{p} \Biggr\} ^{\frac{1}{p}} \\& \quad \quad {}\times \Biggl\{ \sum_{n=1}^{\infty } \bigl[ (n- \eta )^{q(1-\lambda_{2})-1}+(n+ \eta )^{q(1-\lambda_{2})-1} \bigr] b_{n}^{q} \Biggr\} ^{\frac{1}{q}}. \end{aligned}$$

In particular, for $\xi =\eta =0$, $\lambda =1$, $\lambda_{1}=\lambda_{2}=\frac{1}{2}$ in (31) (or for $s=\lambda =c_{1}=1$, $\lambda_{1}=\lambda_{2}=\frac{1}{2}$ in (30)), setting

$$ \theta (m):=\frac{1}{\pi } \int_{m}^{\infty }\frac{u^{-1/2}}{u+1}\,du=O \biggl( \frac{1}{m^{1/2}} \biggr) \in (0,1),\quad m\in \mathbf{N}, $$

we have the following reverse Hardy–Hilbert inequality with the best possible constant *π*:

$$\begin{aligned}& \sum_{n=1}^{\infty }\sum _{m=1}^{\infty }\frac{a_{m}b_{n}}{m+n} > \pi \Biggl[ \sum_{m=1}^{\infty }\bigl(1-\theta (m)\bigr)m^{\frac{p}{2}-1}a _{m}^{p} \Biggr] ^{\frac{1}{p}} \Biggl( \sum_{n=1}^{\infty }n^{ \frac{q}{2}-1}b_{n}^{q} \Biggr) ^{\frac{1}{q}}. \end{aligned}$$

Hence (21) is an extended reverse Hardy–Hilbert’s inequality in the whole plane.

## Conclusions

In this paper, using the weight coefficients, a complex integral formula, and Hermite–Hadamard’s inequality, we give an extended reverse Hardy–Hilbert’s inequality in the whole plane with multiparameters and a best possible constant factor (Theorems 5 and 6). We consider equivalent forms and a few particular cases. The technique of real analysis is very important, which is the key to prove the reverse equivalent inequalities with the best possible constant factor. The lemmas and theorems provide an extensive account of this type inequalities.
